# Myokines and Heart Failure: Challenging Role in Adverse Cardiac Remodeling, Myopathy, and Clinical Outcomes

**DOI:** 10.1155/2021/6644631

**Published:** 2021-01-13

**Authors:** Alexander E. Berezin, Alexander A. Berezin, Michael Lichtenauer

**Affiliations:** ^1^Internal Medicine Department, State Medical University, Ministry of Health of Ukraine, Zaporozhye 69035, Ukraine; ^2^Internal Medicine Department, Medical Academy of Post-Graduate Education, Ministry of Health of Ukraine, Zaporozhye 69096, Ukraine; ^3^Department of Internal Medicine II, Division of Cardiology, Paracelsus Medical University Salzburg, 5020 Salzburg, Austria

## Abstract

Heart failure (HF) is a global medical problem that characterizes poor prognosis and high economic burden for the health system and family of the HF patients. Although modern treatment approaches have significantly decreased a risk of the occurrence of HF among patients having predominant coronary artery disease, hypertension, and myocarditis, the mortality of known HF continues to be unacceptably high. One of the most important symptoms of HF that negatively influences tolerance to physical exercise, well-being, social adaptation, and quality of life is deep fatigue due to HF-related myopathy. Myopathy in HF is associated with weakness of the skeletal muscles, loss of myofibers, and the development of fibrosis due to microvascular inflammation, metabolic disorders, and mitochondrial dysfunction. The pivotal role in the regulation of myocardial and skeletal muscle rejuvenation, attenuation of muscle metabolic homeostasis, and protection against ischemia injury and apoptosis belongs to myokines. Myokines are defined as a wide spectrum of active molecules that are directly synthesized and released by both cardiac and skeletal muscle myocytes and regulate energy homeostasis in autocrine/paracrine manner. In addition, myokines have a large spectrum of pleiotropic capabilities that are involved in the pathogenesis of HF including cardiac remodeling, muscle atrophy, and cardiac cachexia. The aim of the narrative review is to summarize the knowledge with respect to the role of myokines in adverse cardiac remodeling, myopathy, and clinical outcomes among HF patients. Some myokines, such as myostatin, irisin, brain-derived neurotrophic factor, interleukin-15, fibroblast growth factor-21, and growth differential factor-11, being engaged in the regulation of the pathogenesis of HF-related myopathy, can be detected in peripheral blood, and the evaluation of their circulating levels can provide new insights to the course of HF and stratify patients at higher risk of poor outcomes prior to sarcopenic stage.

## 1. Introduction

Heart failure (HF) remains a global public health problem with rapidly increasing prevalence that affects 37 million individuals and more worldwide [1]. Despite the significant achievements in the management of cardiovascular (CV) risk factors and novel therapies of HF with reduced ejection fraction (HFrEF) and stabilized incidence of new cases of predominant HF with preserved ejection fraction (HFpEF) in many countries, morbidity and mortality in patients with both phenotypes of HF continue to be unacceptably high [2, 3]. Being associated with a high risk of hospitalization, HF yields a substantial economic burden for health system and patients' families [4, 5].

Current data for the prognosis of the patients having HFrEF and HFpEF show that the proportion of CV deaths is higher in HFrEF than HFpEF, but the number of non-CV death is higher in HFpEF when compared to HFrEF [6]. These findings are a result of an influence of age, CV risk factors, and several comorbid conditions, such as diabetes mellitus, abdominal obesity, hypertension, chronic kidney disease, and coronary artery disease [7]. Although comorbidity is common in both phenotypes of HF, but it is slightly more severe and occurs more frequently in HFpEF than in HFrEF [8, 9]. There is the assumption that the comorbidities, such as overweight, abdominal obesity, and diabetes mellitus, may alter myocardial structure and impair cardiomyocyte function through several intramyocardial signaling pathways (hypophosphorylation of titin and irisin, cyclic guanosine monophosphate/protein kinase G activity, activation of Janus 1/2 kinases and nuclear factor Kappa B, and suppression of phosphatidylinositol 3-kinase (PI3) kinase/mitogen-activated protein (MAP) kinase/mTOR), which are result of systemic inflammation and thereby cause coronary microvascular endothelial inflammation and oxidative stress [10]. Consequently, substantial reduction of nitric oxide bioavailability and low activity of protein kinase G favors the development of cardiac hypertrophy and increases stiffness of the myocardium due to accumulation of extracellular matrix [11]. Finally, both cardiac hypertrophy and interstitial fibrosis contribute to diastolic abnormalities and the development of HFpEF [12]. In contrast, HFrEF is directly related to sufficient loss of the cardiac myocytes due to necrosis resulting of ischemia, inflammation, and apoptosis that are associated with adverse cardiac remodeling, systemic neurohormonal activation, peripheral vascular effects, skeletal muscle dysfunction, and metabolic abnormalities [13, 14]. However, the impaired physical activity due to muscle weakness, skeletal myopathy, muscle atrophy, and finally cachexia is the attributive factor for HF progression and it is closely associated with increased CV mortality, HF hospitalization, and decrease in the quality of life [15].

The underlying pathophysiological mechanisms of impaired physical activity in HF are abnormal energy metabolism of skeletal muscles, adiposity-related proinflammatory cytokine production, skeletal muscle mitochondrial dysfunction, the transition of myofibers from type I to type II in skeletal muscle, reduction in muscular strength, myocyte apoptosis, and loss of the number of myocytes with shaping of muscle atrophy [16]. Myokines are defined as cytokines that are produced by skeletal muscle myocytes and cardiac myocytes and regulate the crosstalk between skeletal muscle, adipose, and bone tissue [17]. Normally, myokines ensure the molecular adaptations of skeletal muscles to physical exercise and hemodynamic supply acting as regulator of exercise intolerance. The altered myokines' profile is also responsible for metabolic or hormonal derangements in skeletal muscles in HF patients even at early stage of the disease and probably could be a target for the therapy of the disease [18]. The aim of the narrative review is to summarize the knowledge with respect to the role of myokines in adverse cardiac remodeling, myopathy, and clinical outcomes among HF patients.

## 2. Methodology

The bibliographic database of life science and biomedical information MEDLINE, EMBASE, Medline (PubMed), the Web of Science, and the Cochrane Central were searched for English publications satisfying the key words of this study. We used the following key words [heart failure], [cardiac dysfunction], [adverse cardiac remodeling], [cardiac remodeling], [myokines], [myopathy], [cardiac cachexia], [cardiovascular risk], [cardiovascular risk factors], [cardiac biomarkers], [circulating biomarkers], [prognosis], and [clinical outcomes]. All authors independently evaluated each other the quality of the articles, correspondence to the main idea of the study, and constructed the final list of the references. Consequently, strengths and weaknesses of each paper that was selected, as well as unblinded list of the references, were deeply considered by all authors. Final version of references, data for evaluation, and completed proof of the narrative review were approved by all authors.

## 3. Skeletal Muscle Myopathy and HF: The Conventional View

Skeletal muscle myopathy with or without weight loss appears to have a more pronounced significance when compared with weight loss alone with regard to functional capacity, decreased endurance, and quality of life among HF patients [19]. To note, fatigue and muscle weakness can be occurred prior to established diagnosis of skeletal muscle myopathy and even sarcopenic stage of HF, and the altered profile of myokines is deeply discussed as one of the earliest pathophysiological changes of energy metabolism that undoubtedly play a pivotal role in adaptation of skeletal muscles to impair diastolic and pump functions and consequently reduce skeletal muscle perfusion.

**Figure 1:**
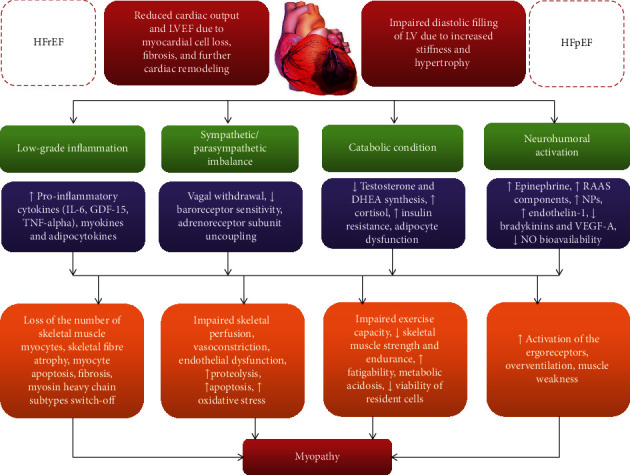
The pathogenesis of the skeletal muscle myopathy in HF. RAAS: renin-angiotensin-aldosterone system; NO: nitric oxide; NP: natriuretic peptides; IL: interleukin; GDF: growth differential factor; TNF: tumor necrosis factor; HFrEF: heart failure with reduced ejection fraction; DHEA: dehydroepiandrosterone; HFpEF: heart failure with preserved ejection fraction.

The pathogenesis of the skeletal muscle myopathy in HF is reported Figure 1. In fact, HF-related skeletal myopathy is characterized by decreased muscle strength, atrophy of fiber I and IIa subtypes, ongoing microvascular inflammation, oxidative stress and damage under metabolic homeostasis, and impaired reparation after muscle injury [20]. Indeed, low perfusion of the skeletal muscles in HF leads to muscle injury that includes ischemia-induced metabolomics (shifted metabolic substrate utilization, altered mRNA expression of insulin-like growth factor (IGF) 1, type 1 receptor (IGF-1R), binding protein 3, lactate accumulation, and acidosis) and mitochondrial (impaired mitochondrial electron transport chain activity, increased formation of reactive oxygen species, and impaired ion homeostasis) abnormalities, which increased expression of the proinflammatory cytokine genes, as well as necrosis and apoptosis of myocytes [21]. Finally, that is associated with myosin heavy chain subtypes switch-off, decreased capillary/fiber ratio, the number of type I fibers, the development of fibrosis, and the loss of the skeletal muscle mass [22]. In addition, systemic inflammation and neurohumoral activation (increased activity of the renin-angiotensin-aldosterone system, sympathetic system, and endothelin-1) lead to decreased bioavailability of nitric oxide and other regulators of vasodilation and angiopoiesis, such as bradykinins and vascular endothelial growth factor, and aggravate endothelial dysfunction, muscle perfusion, and muscle metabolism [17, 23]. Moreover, coexisting adipocyte dysfunction supports systemic inflammation through overproduction of inflammatory cytokines (interleukin- (IL-) 6, tumor necrosis factor- (TNF-) alpha) and induces catabolic state and impairs viability and differential capability of various progenitor cells including endothelial and muscle cell precursors [24]. Yet, impaired baroreceptor sensitivity, vagal withdrawal, and uncoupling of the subunits of beta-adrenoreceptors maintain vasoconstriction and hypoperfusion of the skeletal muscles tailoring the vicious circle in the pathogenesis of the HF-related myopathy.

Because physical endurance, several anthropomorphic features, body mass, and CV risk factors including abdominal obesity and diabetes occurred in male and female in different ways, there is a suggestion that myokine profiles can distinguish in both sexes [25]. Perhaps, expression pattern and signature of circulating myokines would partially explain the complicated crosstalk between skeletal muscle and other tissues, such as WAT, in different genders and muscle phenotypes [26]. Indeed, metabolic signature in Duchenne muscular dystrophy is related to gender and depended on expression of genes, which were widely involved in the pathogenesis of the disease, i.e., matrix metalloproteinase- (MMP-) 9, brain-derived neurotrophic factor (BDNF), adiponectin, persephin, osteomodulin, protooncogene tyrosine-protein kinase receptor Ret, complement decay-accelerating factor, growth differentiation factor 11 (GDF-11), gelsolin, and tumor necrosis factor receptor superfamily member 19L [27, 28]. Although primary causes are significantly different for HF-induced myopathy and Duchenne muscular dystrophy, myokines are engaged in the pathogenesis of cardiac abnormalities for both diseases [28].

## 4. Myokines in HF Myopathy

**Figure 2:**
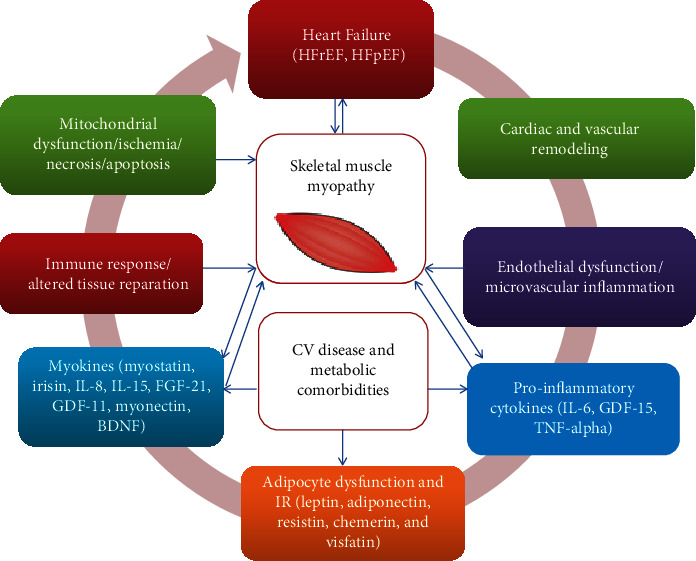
The role of skeletal muscle dysfunction in the pathogenesis of HF. HFpEF: HF with preserved ejection fraction; HFrEF: HF with reduced ejection fraction.

The skeletal muscles enable to release a wide range of the biological active molecules with variable potencies called myokines; the profile of which was found to be altered in HF patients [29]. Although HF-related myopathy has been considered as secondary muscle injury that was associated with low capillary perfusion [30], myokines ensure adaptive metabolic autoregulation of structure and function of skeletal muscles at the early stage of the disease and consequently altered profile of the myokines corresponded to progression of HF and occurrence of sarcopenia and cachexia [31]. Moreover, the periods of acute HF exacerbation and hospitalization are associated with substantial low physical activity. Consequently, impaired synthesis and releasing of myokines lead to the protein metabolic derangements in both the skeletal muscles and myocardium aggravating muscle weakness, physical intolerance, and cardiac dysfunction. In addition, several comorbidities, such as abdominal obesity and diabetes mellitus, coexisting with HF can also alter the profile of myokines including irisin, myostatin, brain-derived neurotrophic factor (BDNF), and growth differential factor-11 (GDF-11) and lead to muscle weakness [32–34]. Interestingly, there was no strong correlation of HF-induced myopathy with left ventricular (LV) ejection fraction (EF) in HFpEF/HFrEF patients, whereas global longitudinal strain was positively associated with the occurrence of the myopathy due to HF regardless of LVEF [35]. Probably, *in situ* cardiac dysfunction is not the only factor contributed to advance of HF-induced myopathy and circulating regulators of energy homeostasis can be promising indicator of HF progression. In this context, primary impairment of the skeletal muscle homeostasis has been speculated as a crucial mechanism in the occurrence and the development of the HF in patients with metabolic diseases predominantly diabetes mellitus and abdominal obesity [36, 37]. In fact, there is vicious circle that corresponds to aberrant skeletal muscle impairments and pathophysiological mechanisms of HF development (Figure 2).

There is evidence for the fact that the wide spectrum of myokines provides controversial actions on skeletal muscle cells and mediates pleiotropic effects. Most of myokines are controlled by muscle contractility function, myogenesis, muscle hypertrophy, and reparation and consequently closely regulate exercise tolerance via intracellular signal pathways including the Janus 1 and 2 kinases/3 and 5 signal transducer and activator of transcription proteins/nuclear factor kappa B, PI3 kinase, and MAP kinase pathways [38]. It is interesting that some potential proinflammatory myokines, such as IL-15 and IL-6, simultaneously provide angiopoietic effects and support proapoptotic impact on myoblasts. It has been found interrelationship between NO-mediated cellular signaling and production of the myokines in skeletal muscle cells [39]. However, hyperemia in skeletal muscle over physical exercise was strongly associated with myokine release [40]. In addition, occurrence of cardiac cachexia in HF is accompanied by crossover changes in the spectrum of the myokines; for instance, there were elevated serum concentrations of myostatin and IL-6 found, whereas isirin, fibroblast growth factor- (FGF-) 21, and myonectin demonstrated a significant decrease in their circulating levels. The serum levels of decorin, BDNF, and GDF-11 were variable and exhibited strong relation to age of the HF patients rather than severity of contractility dysfunction and sarcopenia [41–44]. Finally, myokines influence not just skeletal muscles but also the myocardium and adipose tissue and ensure their autocrine metabolic regulation of energy homeostasis, hypertrophy, reparation, and adaptation of skeletal muscles to physical exercise.

The biological effects and HF-related actions of several myokines are reported Table 1.

### 4.1. Decorin

Decorin is a proteoglycan that is produced by skeletal muscles in a result of stretching and constitutively suppresses the extracellular matrix (ECM) accumulation, particularly type I fibrillar collagen, stimulates angiogenesis and reparation, and negatively regulates inflammation, oxidative stress, and apoptosis [41, 44].

Development of HF was associated with downregulation of decorin expression in the myocardium and consequently increases in activity of matrix metalloproteinase- (MMP-) 2 that corresponded to adverse cardiac remodeling [45]. There is evidence regarding the fact of that decorin interfered with cardiac myocytes and switched off their transcriptome to suppress synthesis of MMP tissue inhibitors and substantially upregulate cardiac fibrosis-associated transcripts including collagen I and III, elastin, lumican, and periostin [46, 47]. In addition, decorin was potentially encouraged in adverse cardiac remodeling by directly inhibiting the transforming growth factor-beta (TGF-beta) pathway and increasing collagen mRNA transcription in the myocardium [48]. As a result of these actions, cardiac myocytes face increased matrix rigidity that lad to diastolic filling abnormality [49]. To sum up, decorin being a natural antagonist of TGF-beta enables to prevent cardiac fibrosis and hypertrophy and improve cardiac function.

### 4.2. Irisin

Irisin is a multifunctional hormone-like active peptide that is produced in abundance by the myocardium and skeletal muscle in response to ischemia, volume overload, inflammation, and physical exercise [50].

Irisin is synthesized as a result of proteolytic cleavage of specific precursor (fibronectin type III domain-containing protein-5—FNDC5) that is expressed on the surface of myocytes. Having numerous autocrine skeletal muscle effects (attenuation of energy expenditure through enhancement of glucose uptake, improvement of oxidative metabolism, and increase in myoblast differentiation), which are ensured by upregulation of the expression of FNDC5, irisin enables to cooperate with white adipose tissue (WAT) to induce its browning by increasing the expression of mitochondrial uncoupling protein 1 (UCP 1) and subsequently activates nonshivering thermogenesis, supports glucose homeostasis, and reduces endothelial function abnormality, insulin resistance, and adipose tissue inflammation [51–53]. Therefore, irisin protects the myocardium against ischemia and reperfusion injury and attenuates the proliferation of the endothelial precursors acting through the AMPK-Akt-eNOS-NO pathway [54, 55]. There is evidence for reduction of cardiomyocyte apoptosis and alleviation of myocardial hypertrophy caused by pressure overload with irisin [56]. Irisin also plays a pivotal role in the control of bone mass with positive effects on cortical mineral density and bone geometry through an interaction with *α*V/*β*5 integrin [57, 58]. In addition, irisin is involved in the process of neurogenesis in the central and peripheral nervous system [59, 60].

Serum levels of irisin were independently associated with Framingham risk profile [61]. The circulating levels of irisin were significantly higher in the normoglycemic patients with metabolic syndrome, but not those who had prediabetes or diabetes mellitus in comparison with healthy volunteers [62]. There is evidence for lower levels of irisin in patients with stable coronary artery disease (CAD) or acute coronary syndrome/myocardial infarction [63–65]. Moreover, decreased serum levels of irisin were noticed to be associated with the presence, severity, and higher SYNTAX score of stable CAD [66, 67]. Interestingly, serum levels of irisin among myocardial infarction patients having HF were reduced when compared with healthy volunteers, but did not differ from those who had no HF [64]. However, there were found positive associations between serum levels of irisin and LVEF [64]. Silvestrini et al. (2019) [68] reported that circulating levels of irisin were significantly higher in HFpEF than in HFrEF patients and did not correlate with homeostasis model assessment of insulin resistance (HOMA-IR) index in both patient cohorts. Irisin levels demonstrated positive correlation with brain natriuretic peptide (BNP) levels and New York Heart Association (NYHA) class of HF and inverse correlation with body mass index (BMI) and catabolic state including cachexia in HFrEF [69]. Yet, FNDC5 expression in skeletal muscles is related to aerobic performance in patients with HFrHF [70, 71]. Thus, irisin plays a protective role in myocardial ischemia, cardiac myocyte apoptosis, and skeletal myopathy preserving energy homeostasis, attenuating mitochondrial function, and regulating muscle atrophy.

### 4.3. Myonectin

Myonectin (also known as erythroferrone) is a myokine, which belongs to the C1q/tumor necrosis factor- (TNF-) related protein (CTRP) family and is upregulated in skeletal muscles by physical exercise and is determined in peripheral blood in elevated concentrations [72]. Circulating levels of myonectin were also strongly regulated by the metabolic state (feeding, obesity, diabetes mellitus, and cachexia), and thereby, myonectin is a conductor between skeletal muscle with lipid homeostasis in liver and WAT [73]. Typically, patients with abdominal obesity, metabolic syndrome, and type 2 diabetes mellitus demonstrate higher circulating levels of myonectin than healthy volunteers [73]. In addition, among patients having prediabetes and diabetes mellitus, serum levels of myonectin correlated positively with waist/hip ratio, percentage of body fat, fasting blood glucose, 2-hour blood glucose after glucose overload, fasting insulin, triglyceride, hemoglobin A1c, and HOMA-IR [74]. Although the primary biological role of myonectin is an increase of free fatty acid uptake by skeletal muscles and contributing to lipid and glucose metabolism in adipose tissue, it has several pleiotropic effects, such as suppression of inflammatory response, protection of ischemia-reperfusion injury, and improvement of endothelial function that are mediated through the S1P/cAMP/Akt-dependent signaling pathway [75, 76]. The development of HF is associated with downregulation in myonectin expression due to inflammatory response, but the pathogenetic role of this myokine in the disease is not fully understood [77].

Thus, myonectin acts as an endurance exercise-induced myokine ameliorating compensatory mechanism against insulin resistance and attenuating acute myocardial ischemic injury by inhibition of apoptosis and suppression of inflammation in the myocardium.

### 4.4. Fibroblast Growth Factor 21

Fibroblast growth factor (FGF-21) is a multifactor protein, which is produced by several organs and engaged in the autocrine/paracrine regulation of fatty acid oxidation, energy expenditure, glucose homeostasis, and the functions of somatotropic axis and hypothalamic-pituitary-adrenal pathway [78, 79]. This peptide is highly expressed in the myocardium, pancreas, WAT, liver, brain, and kidney, but not constitutively in the skeletal muscles [80]. In addition, FGF-21 is induced in situations of muscle stress, particularly mitochondrial dysfunction. The beneficial effects of FGF21 include weight loss, improvement of glycemia and lipotoxicity, browning WAT, suppression of inflammation and oxidative stress, counteracting water intake, and blood pressure elevation [81–84]. FGF-21 induced the expression of genes, which encode proteins involved in antioxidative pathways, such as mitochondrial uncoupling proteins (Ucp2 and Ucp3) and superoxide dismutase-2 (Sod2) and reduced ROS production [85, 86]. In addition, FGF-21 prevented the development of cardiac hypertrophy by activating MAPK signaling through the activation of FGF-R1c with *β*-klotho as a coreceptor [87, 88].

In a failing heart, FGF-21 exerts protective effects, preventing the development of cardiac hypertrophy and ischemic injury via the Sirt1 (sirtuin-1) pathway [85, 89]. Patients with multivessel CAD and type 2 diabetes mellitus have revealed decreased expression of FGF-21 in the myocardium [90]. Among patients with HFrEF and HFpEF, serum levels of FGF-21 were positively associated with echocardiographic parameters of diastolic function, LV end-diastolic pressure, and NT-pro-BNP levels [88–90], as well as with IL-6 levels and lower skeletal muscle mass [91]. Conflicting results in the presence of the strong correlation between serum levels of FGF-21 and NT-pro-BNP are explained by adjustment of the data for cardiac cachexia [91, 92]. There was strong correlation between circulating levels of FGF-21 and NT-pro-BNP in HFrEF patients without cardiac cachexia, but no correlation between these biomarkers was noticed in those who had HFrEF with cardiac cachexia [93]. Probably, some cardioprotective effects of sodium-glucose cotransporter 2 inhibitors (SGLT2i) may be related to their ability to induce the FGF-21/SIRT1 pathway and thereby stimulate endogenous reparation [94, 95].

### 4.5. Myostatin

Myostatin is established as a negative regulator of skeletal muscle mass that is upregulated in the myocardium of HF [96, 97]. Myostatin belongs to the transforming growth factor-*β* (TGF-*β*) family and its main biological function comes down to the inhibition of skeletal muscle growth and prevention of insulin resistance [98]. In the physiological condition, myostatin is predominantly expressed in skeletal muscle, while small basal expression was also noticed in the myocardium and WAT.

The development of the HF is associated with increased expression of myostatin in the myocardium, skeletal muscles, and WAT, and elevated levels of the peptide were discovered in the peripheral blood [99]. Myostatin interacts tightly with insulin-like growth factor I (IGF-I) and enables to stimulate the expression of regulator of G-protein signaling 2, a GTPase-activating protein, which restricts the Gaq and Gas signaling pathway and thereby protects against ischemic/reperfusion injury and HF development [100]. Muscle myopathy and sarcopenia are related to overexpression of myostatin that acts as powerful activator of the Smad2/3 pathway and thereby stimulates the proteasomal and the autophagic-lysosomal capabilities [99–101]. On the other hand, overexpression of myostatin in the myocardium caused interstitial fibrosis and myocyte loss via activation of the TAK-1-MKK3/6-p38 signaling pathway, and thereby, these findings did not support the idea about protective abilities of myostatin [102]. Yet, myostatin has demonstrated an ability to modulate myosin heavy chain isoform (I MyHC isoform) shift in skeletal muscle [103]. However, being a strong predictor of frailty, disability, and mortality sarcopenia occurs in patients with HFrEF in results of abundant molecular mechanisms including Smad2/3 signaling [101, 104]. Probably, these controversies in the protective ability of myostatin relate to etiology (ischemic or nonischemic) of HF [105, 106].

Serum levels of myostatin were found to be higher in chronic HF patients than in healthy volunteers which positively correlated with biomarkers related to HF severity [107–109]. In addition, there is evidence for significant decreasing of serum levels in HF patients [110]. However, the results regarding the association between myostatin levels, HF severity, and other HF biomarkers such as NT-proBNP/BNP are conflicting. Chen et al. (2019) [107] found strong associations between myostatin and severity of adverse cardiac remodeling, NYHA classes of HFrEF and LVEF, while Zamora et al. (2010) did not notice these relations in HFrEF patients [111]. Thus, myostatin is involved in adverse cardiac remodeling and the evaluation of its circulating levels appears to be promised to predict the course of HF and also to guide a risk stratification of HF patients [110].

### 4.6. Brain-Derived Neurotropic Factor

Brain-derived neurotropic factor (BDNF) is a neuronal growth factor that plays a pivotal role in the maintenance of the nervous system, the development of depression and behavior disorders, cardiac reparation, and skeletal muscle energy metabolism [112, 113]. BDNF is produced by a wide spectrum of the cells including cardiac myocytes, skeletal muscles, smooth muscle cells, and mature and progenitor endothelial cells [112, 114]. Typically, BDNF is considered as multifunctional protein with organ protective capabilities, which is the synthesis in the result of tissue damage (ischemia, hypoxia) and an impact of proinflammatory cytokines (IL-1*β*, IL-6, and TNF-alpha) and acts through the c-Jun N-terminal kinase pathway [114–116]. It has been suggested that physical exercise and strength exercise are able to ensure an effective cardiometabolic protection through increasing BDNF serum levels [117]. There is evidence for the fact that BDNF was found to be a powerful metabolic regulator of myoblast activity, and thereby, this protein is engaged in the endogenous reparation of the skeletal muscle and myocardium [116, 117]. In addition, BDNF is involved in the regulation of glucose and lipid metabolism [118].

There is Val66Met polymorphism (rs6265) of BDNF gene, which has been associated with altered circulating levels of BDNF and corresponds to several neuropsychiatric disorders, regional structural brain changes, and cardiac and vascular protection against hypoxia and ischemia [119–121]. In fact, obesity, metabolic syndrome, type 2 diabetes mellitus, and CV diseases including HF were associated with decreased serum levels of BDNF [122–124]. In contrast, patients having acute coronary syndrome (ACS) and ST segment elevation myocardial infarction had higher circulating levels of BDNF than healthy volunteers and strongly predicted acute HF development [125], while there is evidence regarding that BDNF levels can be reduced in patients having ACS [126]. Moreover, in animal model of HF and among patients with HFrEF, low levels of BDNF were associated with reduced physical activity and a risk of HF-related myopathy [127–131]. In addition, low levels of BDNF predicted a risk of cognitive dysfunction among HFrEF patients [132] and poor clinical outcomes [124, 125, 131, 133]. The serum BDNF levels may be a useful surrogate biomarker of increased CV risk, the HF-related myopathy, and adverse prognosis in patients having HF [134].

### 4.7. Interleukin-8

IL-8 (also known as chemokine CXCL8) belongs to the CXC chemokine family [135]. It is produced by mononuclears, phagocytes, adipocytes, epithelial cells, endothelial cells, and mesenchymal cells exposed to various inflammatory stimuli [136]. IL-8 acts as paracrine trigger of macrophage migration, neutrophil chemotaxis, and differentiation and proliferation of profibrogenic mesenchymal progenitor cells [137, 138]. Interestingly, skeletal muscle fibers express IL-8 mRNA, which is regulated by muscle contraction [139]. Thus, IL-8 can partially be considered as a myokine.

Although IL-8 have shown to be predictive for CV events in several studies, its role as clinical biomarkers for HF is unclear [140–142]. Elevated levels of IL-8 were noticed in the patients with ACS and acute HF [143, 144]. There was strong correlation between serum levels of circulating IL-8 and adverse clinical outcome and cardiac remodeling after STEMI [142]. However, there are conflicting reports regarding the role of IL-8 in inducing cardiac dysfunction in HF [145–147]. Indeed, delayed expression of IL-8 in the myocardium after revascularization in STEMI was associated with a high risk of cardiac dysfunction and the development of chronic HF regardless of the presence of traditional CV risk factors [146]. On the other hand, several comorbidities including type 2 diabetes mellitus and abdominal obesity were able to aggravate adverse cardiac remodeling after completed reperfusion through microvascular inflammation [147]. In addition, there were no favorable effects on CV events observed in the large clinical trials of rheumatoid arthritis patients treated with anticytokine therapy (IL-1*β* inhibition, IL-1 receptor antagonists, IL-6 receptor antagonists, or TNF inhibition) [148]. Finally, the role of IL-8 in HF development and progression remains uncertain.

### 4.8. Interleukin-15

IL-15 is pleiotropic proinflammatory cytokine with structural similarity with IL-2, which predominantly exerts anabolic and tissue protective effects by decreasing cardiac myocyte apoptosis, mobilization of endothelial and mesenchymal progenitor cells, reduction of oxidative stress, and improvement of myocardial function [149]. On hypoxia condition, cardiac myocytes express appropriate IL-15 receptor, by which IL-15 protects the myocardium against injury [149]. IL-15 activates signaling by the *β* and common *γ* (*γ*c) chain heterodimer of the IL-2 receptor and thereby supports survival and proliferation of natural killer cells and suppresses oxidative stress [150]. On the other hand, IL-15 maintains chemotaxis of the natural killer cells and their adhesion on endothelium [151]. In contrast to IL-8, IL-15 is a growth factor that is highly expressed in skeletal muscle and exerts muscle hypertrophy through specific receptor (IL-15R) [152, 153]. Despite IL-15 has been revealed as having sufficient anabolic impact on skeletal muscle both *in vitro* and *in vivo*, it plays a crucial role in reducing mass of WAT [139].

IL-15 is upregulated in some CV diseases, such as myocardial infarction, HF and atherosclerosis [154]. In addition, IL-15 was found a regulator of fractalkine (FKN)-CX3CR1 chemokine signaling system, which is involved in the acceleration of atherosclerosis and promoting smooth muscle cell proliferation [155]. There is evidence of the fact that IL-15 has direct cytotoxic impact on endothelial cells and their precursors and thereby induces endothelial dysfunction and microvascular inflammation. Indeed, IL-15 activates antigen-presenting cells (APCs), such as dendritic cells, macrophages, and CD8(+) T cells and acts as a trigger of apoptosis of endothelial cells via caspase activation and loss of mitochondrial membrane potential [156, 157]. Thus, IL-15 is able to support inflammatory infiltration of the myocardium and directly induce myocardial injury. Additionally, IL-15 exerts specific endocrine effects on WAT, stimulates synthesis of adipocytokines, and consequently maintains adipocyte tissue oxidation and inflammation [158]. Finally, acting through the regulation of adipocytokine synthesis, IL-15 indirectly modifies IR of skeletal muscles and attenuates HF-related myopathy [159, 160].

Low circulating levels of IL-15 were found in younger and older people with sarcopenia without known HF [161, 162]. A clinical study has shown that IL-15 gene polymorphisms were susceptible biomarkers for development of subclinical atherosclerosis and CAD [163]. Patients with HF have demonstrated altered profile of several cytokines including IL-15; the levels of which were noticed to be dramatically increased [164]. However, circulating levels of IL-15 were not associated with ischemia-induced adverse cardiac remodeling and poor clinical outcomes [164], but in nonischemic HF, patients there found a correlation between IL-15 myocardial expression and a risk of sudden death and HF-related events [165].

To sum up, the results of preclinical and clinical studies for the integral role of IL-15 as a trigger of adverse cardiac remodeling and HF-related myopathy have been noticed inconclusive. Although the natural killer cell receptor/IL-15 signaling pathway contributes to progressive inflammatory muscle destruction and myopathy [166], whether this molecular mechanism is essential for regulation of HF-related myopathy is not fully clear. Thus, muscle-derived IL-15 appears to have important roles in metabolism of both the myocardium and skeletal muscles, and exercise plays a role in the interplay between WAT modification and inflammation, but its value in HF-related myopathy remains to be poorly understood [167, 168].

### 4.9. Growth Differential Factor-11

GDF-11 belongs to the superfamily of transforming growth factor-beta and is widely expressed in several tissues including the myocardium and skeletal muscles [169]. GDF-11 reverses age-related cardiac hypertrophy, improves muscle regeneration and angiogenesis, maintains differentiation of progenitor cells, and protects against myocardial ischemia and reperfusion injury by activation of Smad2/3 signaling [170–172]. Nevertheless, in animal model of HF, GDF-11 stimulated oxidative stress, potentiated apoptosis, and induced tissue injury by upregulating Nox4 in H9C2 cells (cardiomyoblast cell line derived from embryonic rat heart tissue) and the production of reactive oxygen species in a result of modulation of NADPH oxidases [173].

GDF-11 is upregulated in the myocardium and skeletal muscles in patients with CAD and HF [174, 175] and acts on skeletal muscles inducing low physical tolerance and muscle weakness [176]. It has been suggested that GDF-11 induces specific genes called astrogenes, which induce the ubiquitin-proteasome system, leading to protein degradation in skeletal muscles and mitochondrial dysfunction [176]. Finally, GDF-11 is discussed as a factor that contributes to disease progression and loss of skeletal muscle mass [177]. In contrast, there is a suggestion that elevation of GDF-11 is associated with cardioprotection, because patients having stable CAD and elevated levels of GDF-11 levels were associated with lower risk of CV events and death [178]. Thus, GDF-11 had cardioprotective activities and probably plays a pivotal role in prevention of HF-related myopathy and sarcopenia.

Overall, the development of HF is associated with upregulation of myostatin and IL-8 and downregulation of irisin, myonectin, FGF-21, BDNF, and IL-15.

### 4.10. SPARC

SPARC (secreted protein acidic and rich in cysteine), also called osteonectin, is an extracellular collagen-binding matrix protein responsible for cell function as well as cell-matrix interactions [179]. In numerous studies, SPARC was shown to act as a potential mediator of collagen deposition and collagen assembly [180]. Moreover, it was also reported to be involved in cell migration and proliferation as well as tissue repair [181]. The secretion of SPARC is known to be induced through physical exercise, stress, and tissue damage and can be found throughout all body tissues [181, 182]. In mouse models with an inhibited SPARC expression, a reduction in organ fibrosis in the lung, heart, skin, liver, and eye was reported in response to fibrotic stimuli [183]. Similarly, reduced levels of SPARC are associated with osteogenesis imperfecta [184]. Moreover, SPARC is also involved in different malignancies [185, 186]. In the heart, SPARC is expressed by endothelial cells and fibroblasts and by cardiac myocytes to some extent [187]. It is not only upregulated in response to cardiac injury and in areas of cardiac remodeling but also in response to pressure overload [188]. In a mouse model of pressure overload, SPARC was observed to play a pivotal role in in the deposition of insoluble collagen deposition, thus contributing to myocardial stiffness [189]. In the same study, macrophages were identified as possible source for increased SPARC levels in response to pressure overload [190]. Apart from cardiac injury and pressure overload, SPARC was also reported to be connected to the age-dependent increase in left ventricular stiffness [179, 190]. Contrary, SPARC was shown to be increased after acute MI while a temporal relation to scar formation was evident [191, 192]. Similarly, SPARC inactivation leads to an increase in cardiac rupture and dysfunction after acute myocardial infarction in a mouse model [193]. Vice versa, overexpression of SPARC after myocardial infarction showed a cardiac and vascular protective effect [194]. In this regard, an increase in Smad2 phosphorylation was suggested [195]. Moreover, studies have proposed a positive inotropic effect of SPARC in the heart [196]. Thus, given its role in collagen deposition and assembly, the role of SPARC in the heart might have to be interpreted in clinical context. While it may have a positive effect with regard to acute tissue damage, chronically elevated levels of SPARC seem to have a negative effect especially with regard to cardiac remodeling and endothelial integrity. However, further studies on SPARC are warranted to clarify its role in cardiac injury and remodeling to further extent.

## 5. Myokines and Heart Failure-Related Clinical Outcomes

There is a large body of conflicted evidence for predictive values of several myokines for adverse clinical outcomes predominantly in HFrEF [197]. Serum irisin levels were found to be higher in acute HF patients deceased in 1-year follow-up [198]. In addition, there was a close positive correlation between elevated levels of irisin and CV clinical outcomes after myocardial infarction regardless of HF presence [199]. Moreover, irisin has demonstrated better prediction for MACEs to NT-proBNP [199]. Collectively, elevated serum levels of irisin were powerful predictive biomarker for 1-year all-cause and CV mortality in acute and chronic HF patients. Higher circulating levels of FGF-21 were also associated with a high mortality rate, but not CV events in patient with ESRD at a risk of HF [200]. Myostatin was found to be an independent predictor of mortality in HF patients and rehospitalization due to HF progression [107]. Decreased serum levels of BDNF were significantly associated with adverse outcomes in HF patients [124, 125]. There is a large body of evidence regarding the fact that elevated levels of some SPARC proteins, such as osteonectin and osteopontin, have demonstrated a strong association with poor long-term HF-related outcomes including death, and a risk for recurrent hospitalization due to HF among patients with normal body mass, overweight, and obesity [201, 202]. However, there was no finding that osteonectin predicted cardiac cachexia and poor clinical events in patients with HF-related myopathy. The discovery of exact molecular pathways that correspond to the link between myokines and HF outcomes remains uncertain and requires being clearly elucidated in the future [18]. However, the idea regarding that the myokines could be new biological target to point-of-care therapy in HF with various phenotypes is promising especially among HF patients with metabolic comorbidities.

## 6. Perspectives in the Future

Myokines are predictive biological markers that are independently associated with an increased risk of HF-related myopathy and cachexia, while their role in the prediction of adverse cardiac remodeling and risk stratification of clinical outcomes requires thorough investigation in the large clinical trials. Another direction for studies in the future is a modification of myokines' profile in a result of aerobic and interval isometric physical exercise. Because HF-related myopathy is an established predictor of poor clinical prognosis and physical exercise has been determined to be predictably valued, the monitoring of serum levels of myokines could be attractive to stratify HF patients at higher risk of the progression of the myopathy. In addition, myokines can be useful to determine whether the physical exercises are adequate. Therefore, myokines can be targets for the personified therapy of HFrEF and HFpEF to prevent HF-related myopathy and cardiac cachexia. In this context, new anticytokine drugs, such as anti-IL-17 anti-IL-23, could be investigated with this purpose.

## 7. Conclusion

Altered circulating signature of myokines is noticed at the early stage of HF occurrence and was associated with the adverse cardiac remodeling, diastolic filling abnormalities, reduced systolic function and progression of skeletal muscle myopathy. Myokines are not only involved in the pathogenesis of skeletal muscle myopathy but also they could provide new insights to the course of HF and stratify patients at higher risk of poor outcomes prior to sarcopenic stage. Although changes in peripheral blood concentrations of several myokines reflect altered metabolic homeostasis in connection with advance in HF, there is limiting strong evidence regarding independent predictive ability of myokines' signature for mortality and HF-related outcomes and superiority these novel biomarkers to traditional circulating cardiac biomarkers during face-to-face comparisons. Irisin, BDNF, FGF-21, and probably osteonectin are the most promising biomarkers of HF-related myopathy and cachexia, while their role in the prediction of adverse cardiac remodeling and poor outcomes requires to be elucidated in the future.

## Figures and Tables

**Table 1:** Biological effects and HF-related actions of myokines.

Name of myokine	Affiliation	Origin of myokines	Biological action	HF-related actions	References
Decorin	Proteoglycan	Skeletal muscles, fibroblasts, vascular endothelial cells, cardiac myocytes, and smooth muscle cells	↓ Accumulation of ECM, ↑ cell differentiation, ↑ proliferation, and ↓ apoptosis	*Downregulated in HF* ↓ Cardiac hypertrophy, ↑ cardiac fibrosis	[41–49]
Irisin	Muscle tissue-secreted peptide FNDC5	Skeletal muscles, myocardium	↑ Expenditure, ↑ oxidative metabolism, ↑ myoblast differentiation, ↑ glucose uptake	*Downregulated in HF* ↓ Tolerance to physical exercise, ↑ skeletal muscle hypotrophy	[50–71]
Myonectin	CTRP15	Skeletal muscles, adipose tissue	↑ Oxidation of free fatty acid, ↑ oxidative metabolism, ↑ myoblast differentiation, ↑ glucose uptake	*Downregulated in HF* ↑ Skeletal muscle hypotrophy	[72–77]
FGF-21	FGF super family	Cardiac myocytes, pancreas, adipose tissue, liver, brain, and kidney	↑ Glucose uptake and protein synthesis in skeletal muscle, ↓ lipolysis in WAT, ↑ browning of WAT	*Downregulated in HF* ↑ Skeletal muscle mass, ↓ IR, ↑ exercise tolerance	[78–94]
Myostatin	TGF-*β* superfamily	Cardiac myocytes, skeletal muscles	↑ Skeletal muscle fiber-type switches, ↓ fast myosin heavy-chain expression, ↓ differentiation of myoblasts, ↑ ubiquitin-proteasomal activity in myocytes and ILGF-PKB pathway	*Upregulated in HF* ↑ Skeletal muscle hypotrophy, ↑IR, ↑ autophagy, ↑ muscle weakness, ↓ exercise tolerance	[95–111, 203]
BDNF	Neurotrophin family	Cardiac myocytes, skeletal muscles, smooth muscle cells, endothelial cells, astrocytes	↑ Myoblast proliferation, ↑ neurogenesis, ↑ angiogenesis, ↑ vascular reparation	*Downregulated in HF* ↑ Tolerance to physical exercise	[112–134]
IL-8	Cysteine-X-cysteine family of chemokines	Mononuclears, phagocytes, adipocytes, epithelial cells, endothelial cells, and mesenchymal cells	↓ Glucose disposal, ↑ IR	*Upregulated in HF* ↓ Skeletal muscle energy metabolism	[135–147]
IL-15	Pleiotropic cytokine with structural similarity with IL-2	Cardiac myocytes, mononuclear phagocytes	Anabolic effect, ↓ oxidative stress	*Downregulated in HF* ↑ Tolerance to physical exercise, ↑ skeletal muscle mass, ↓ WAT, ↓ apoptosis of cardiac myocytes and myoblasts	[149–168]
GDF-11	TGF-*β* super family	Skeletal muscle, neural stem cells, and cardiac myocytes	↓ Differentiation of myoblasts, angiogenesis, and neovascularization	*Downregulated in HF* ↓ Physical endurance, ↑ skeletal muscle hypotrophy and weakness	[169–178]
Osteonectin	SPARC protein	Cardiac myocytes, skeletal muscles, adipose tissue, bones, mucosa, vasculature, kidney, liver	Potential mediator of collagen deposition and extracellular matrix remodeling	*Upregulated in HF* Predictor of poor HF outcomes, ↑ cardiac contractility and reparation at early stage, ↓ cardiac myocyte survival and vascular integrity at late stage	[179–196]

FGF-21: fibroblast growth factor-21; TGF-*β*: transforming growth factor-beta; IR: insulin resistance; ILGF-PKB: insulin-like growth factor-protein kinase B; WAT: white adipose tissue; GDF-11: growth differentiation factor-11; ECM: extracellular matrix; SPARC: secreted protein acidic and rich in cysteine.

## Data Availability

The manuscript is narrative review and dataset was not generated.

## Abbreviations

BDNF: Brain-derived neurotrophic factor
CV: Cardiovascular
ECM: Extracellular matrix
EF: Ejection fraction
FGF-21: Fibroblast growth factor-21
GDF-11: Growth/differential factor-11
HF: Heart failure
HFpEF: Heart failure with preserved ejection fraction
HOMA-IR: Homeostasis model assessment of insulin resistance
HFrEF: Heart failure with reduced ejection fraction
IGF: Insulin-like growth factor
IL: Interleukin
LV: Left ventricle
MAPK: Mitogen-activated protein kinase
NO: Nitric oxide
NPs: Natriuretic peptides
RAAS: Renin-angiotensin-aldosterone system
TNF: Tumor necrosis factor.
